# A Qualitative Review of Balance and Strength Performance in Healthy Older Adults: Impact for Testing and Training

**DOI:** 10.1155/2012/708905

**Published:** 2012-01-23

**Authors:** Urs Granacher, Thomas Muehlbauer, Markus Gruber

**Affiliations:** ^1^Institute of Sport Science, Friedrich Schiller University Jena, Seidelstra*β*e 20, 07749 Jena, Germany; ^2^Department of Sports Science, University of Konstanz, 78434 Konstanz, Germany

## Abstract

A continuously greying society is confronted with specific age-related health problems (e.g., increased fall incidence/injury rate) that threaten both the quality of life of fall-prone individuals as well as the long-term sustainability of the public health care system due to high treatment costs of fall-related injuries (e.g., femoral neck fracture). Thus, intense research efforts are needed from interdisciplinary fields (e.g., geriatrics, neurology, and exercise science) to (a) elucidate neuromuscular fall-risk factors, (b) develop and apply adequate fall-risk assessment tools that can be administered in clinical practice, and (c) develop and design effective intervention programs that have the potential to counteract a large number of fall-risk factors by ultimately reducing the number of falls in the healthy elderly. This paper makes an effort to present the above-raised research topics in order to provide clinicians, therapists, and practitioners with the current state-of-the-art information.

## 1. Introduction

Demographic change affects western industrialized countries in terms of large increases in the number of senior citizens [1]. One serious concern of industrialized countries is that a greying society will undermine the sustainability of the public health care system since per capita health expenditures are approximately 5.5 times higher for people older than 75 years of age than for those aged 25 to 34 years [2]. A major reason for high medical treatment costs in the elderly is an increased incidence rate for falls and fall-related injuries [3, 4]. Prospective studies indicate that 30% to 60% of community-dwelling older adults fall each year [5–9]. Age, functional impairment, and disability are important factors that contribute to an even increased risk of falling [10]. Approximately 5% to 10% of falls result in serious injuries such as fractures (e.g., femoral neck fractures), head traumata, or joint dislocations requiring hospitalization [10]. Fall-related injuries cause restricted mobility and functional decline in elderly individuals. In fact, 25% to 75% of elderly fallers who sustained a femoral neck fracture do not regain their prefracture level of functional mobility [11]. Further, the medical treatment of fall-related injuries lays a high financial burden on public health care systems. In Germany, total annual costs related to femoral neck fractures amounted to 2.77 billion Euros in 2004. Due to population aging, costs of femoral neck fractures may increase to 3.85 billion Euros in 2030 [4].

The aetiology of falls is generally considered to be multifactorial including extrinsic (e.g., loose rugs, lighting, obstructed walkways) and intrinsic (e.g., muscle weakness, gait and balance disorders) circumstances [12]. In a systematic literature review, Rubenstein and Josephson [12] reported that the above mentioned intrinsic circumstances (i.e., muscle weakness, gait and balance disorders) are the second most common cause for falls in older adults. Muscle weakness induces reduced levels of strength, particularly of the lower extremities [13], and is thus responsible for a performance decrement in activities of daily living (e.g., climbing stairs) [14]. Further, the ability to generate force rapidly declines more precipitously in advancing age than maximal strength [13, 15] and is, in a fall-threatening situation, more relevant for preventing a fall than the capacity to produce maximal strength [16, 17]. Gait and balance disorders in older adults are specifically manifested in an impaired ability to compensate for stance/gait perturbations (i.e., reactive balance) as well as in a compromised capacity to stand/walk (i.e., steady-state balance) particularly while concurrently performing cognitive/motor interference tasks [15, 18]. These so-called multitask situations occur frequently during everyday life. For example, an elderly woman carries a tray with a filled cup from the kitchen to the living room while talking to somebody. It was frequently reported that deficits in reactive and steady-state balance performance put older adults at an increased risk of falling [19, 20].

From a fall-preventive point of view, it is important to know whether there is a relationship between variables of strength and balance. Given the high prevalence of falls in older adults, findings on potential associations between variables of muscle strength and balance could provide scientific rationales to fall-risk assessment and to the development of specifically tailored fall-prevention programs in seniors. Thus, the objectives of this paper are to describe and discuss

- age-related effects on strength/power and balance performance,
- potential associations between measures of muscle strength/power and balance performance,
- the resulting consequences for fall-risk assessment and for fall-preventive intervention programs.

## 2. Age-Related Effects on Strength/Power and Balance Performance

### 2.1. Age-Related Effects on Measures of Strength/Power

Biologic aging as well as physical inactivity results in decreases in maximal isometric, concentric, and eccentric force, rate of force development (RFD) as well as muscle power [13, 15, 21]. More specifically, the capacity to generate force rapidly (i.e., RFD, muscle power) declines at a faster rate than the ability to produce maximal strength [13, 15].

#### 2.1.1. One Repetition Maximum (1RM)

Petrella et al. [21] investigated 1RM strength of the knee and leg extensors in a cohort of young (age 20 to 35 years) and elderly healthy men and women (age 60 to 75 years). The authors observed that the older adults showed significantly lower knee (men: 41%; women: 29%) and leg extensor strength (men: 29%; women: 17%) compared to their younger counterparts. Further, knee and leg extensor strength declined more rapidly in men compared to women. 

In another study, Häkkinen et al. [22] found that maximal bilateral concentric leg extensor strength (1RM) already differed significantly between middle-aged (age 35 to 45 years) and older healthy men and women (age 62 to 78 years). This result was confirmed by Izquierdo et al. [23] who provided percentage rates of differences in concentric 1RM strength between middle-aged (mean age 42 years) and older healthy men (mean age 65 years). Elderly men exhibited significantly lower 1RM half squat (14%), 1RM knee extension (27%), and 1RM bench press (21%) strength. These results came along with higher antagonist muscle activations during dynamic knee extension actions in the older as compared to the middle-aged men. In addition, using the ultrasound technique, significantly greater muscle cross-sectional area of the m. quadriceps femoris was found in the middle-aged adults [23]. 

Age-related differences in 1RM strength are not only present between young/middle-aged and older adults but also between old and older adults. In fact, Lamoureux et al. [24] reported significant differences between old (mean age 63 years) and older healthy adults (mean age 76 years) in concentric 1RM of the leg extensors (46%), the leg curls (42%), the hip extensors (52%), the hip flexors (42%), the hip adductors (56%), the hip abductors (59%), and the plantar flexors (65%). 

In summary, these findings indicate that maximal concentric lower extremity strength is reduced in old compared to middle-aged and young healthy adults and that the most severe losses occur in adults above the age of 75 years. Lower muscle volume as well as increased antagonist muscle activity appears to be responsible for the reduced levels of maximal concentric strength in older adults.

#### 2.1.2. Maximal Isometric Strength (MIS) and Rate of Force Development (RFD)

In two early studies, Asmussen and Heeboll-Nielsen [25] and Larsson et al. [26] observed that isometric muscle strength developed in an inverted U-shaped curve across the lifespan. More specifically, maximal strength of the quadriceps increased up to the third decade, remained almost constant to the fifth decade, and then decreased with increasing age [26].

**Figure 1:**
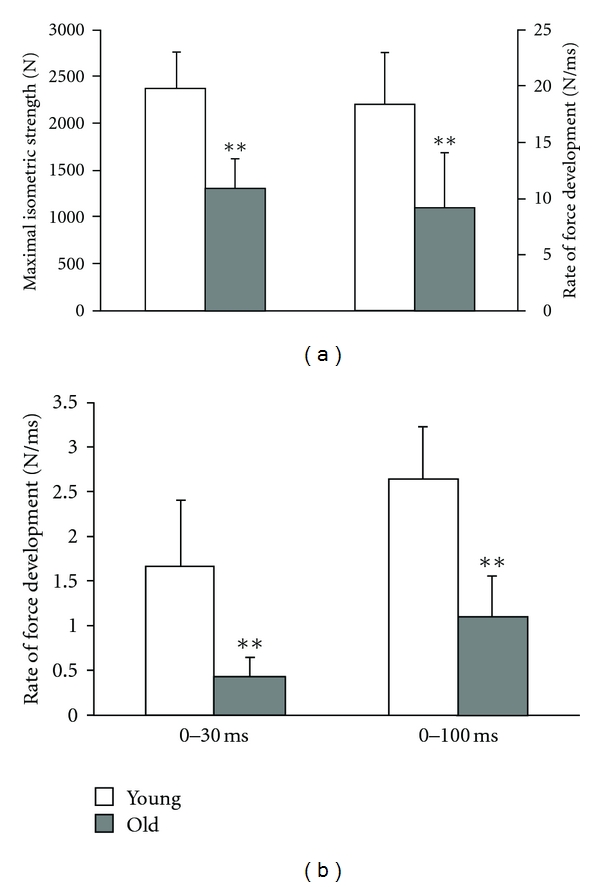
Age-related differences in strength. (a) Maximal isometric strength and rate of force development. (b) Rate of force development over time intervals of 0–30 ms and 0–100 ms. Young different from old: **P < 0.01. Adapted from Granacher et al. [15].

Recently, Granacher et al. [15] investigated MIS and RFD in young (mean age 27 years) and elderly healthy men (mean age 67 years) with special emphasis on the early part of the force-time curve. The authors found that MIS and RFD was significantly lower in old compared to young men (MIS: 45%; RFD: 50%) (Figure 1(a)). Age-related differences were even more prevalent in the early part of the force time curve. In fact, mean slope of the force-time curve over the time intervals 0–30 ms (RFD_30_) and 0–100 ms (RFD_100_) was significantly lower in elderly compared to young men (RFD_30_: 76%; RFD_100_: 59%) (Figure 1(b)). This finding was accompanied by significant reductions in activities of lower extremity muscles (i.e., m. soleus, m. vastus medialis) in the elderly subjects. 

Macaluso et al. [27] found similar results for MIS of the knee extensors/flexors in young (mean age 23 years) and elderly healthy women (mean age 69 years). The older women were on average 43% weaker than the young women in MIS of the knee extensors and 47% weaker in MIS of the knee flexors. Further, activity of the knee extensors and flexors was significantly lower in the old compared to the young women. In addition, muscle contractile volume measured by magnetic resonance imaging was significantly reduced in the older women, both in the knee extensors and flexors. 

Thelen et al. [28] investigated MIS and RFD of the plantar and dorsiflexors in young (age 19 to 29 years) and elderly (age 65 to 86 years) healthy men and women. The older adults were significantly weaker than the younger adults in MIS and RFD of the dorsiflexors (MIS: men 14%, women 21%; RFD: men 25%, women 32%) and the plantarflexors (MIS: men 24%, women 32%; RFD: men 29%, women 36%). 

Samuel and Rowe [29] analysed MIS of the knee and hip joints at 3 positions through the joint range in 3 age groups of healthy older adults (i.e., 60–69 years, 70–79 years, ≥80 years). MIS of the knee and hip joints decreased with increasing age at all the three joint positions. The overall moments at knee and hip joints were approximately 20% lower when comparing those in the 80s with the 60s age group. The overall moments at knee and hip joints decreased by approximately 20% when muscle strength of those in the 80s was compared with the 60s age group. 

In summary, maximal and particularly explosive force production under isometric conditions is significantly lower in old and especially the oldest old adults (≥80 years) compared to young adults. Muscular (i.e., loss in muscle contractile volume) as well as neural factors (i.e., reduced neural drive to activate muscles) account for the age-related reductions in MIS and RFD.

#### 2.1.3. Muscle Power

In an early study, Bosco and Komi [30] analysed the average mechanical power output during SJs in a population ranging in age from 4 to 73 years. The authors observed that peak power increased from childhood to reach peak values between 20 and 30 years. The age-related decline in SJ performance already starts between the ages of 29 to 40 years and it accelerates above 71 years of age [30].

In a more recent study, McNeil et al. [31] found a 25% decrease in power of the dorsiflexors (isotonic contractions) between the third and seventh decade of life. This reduction was doubled in the next two decades, so that men in their ninth decade of life produced 60% less power than young men (mean age 26 years). Similar findings were reported by Skelton et al. [13] who investigated maximal isometric knee extensor strength and leg extensor power in a cohort of 65- to 89-year old men and women. The averaged cross-sectional data across the age range of 65 to 89 years indicate that maximal strength declines at an annual rate of 1.5% and power at a rate of 3.5% [13].

In a sophisticated approach, Petrella et al. [21] determined peak concentric knee extensor power across a load spectrum that included 5 submaximal loads relative to maximum isometric voluntary contraction force (i.e., 20, 30, 40, 50, 60% of maximum isometric force) in a cohort of young (age 20 to 35 years) and elderly healthy men and women (age 60 to 75 years). During the tests, the concentric phase was performed as rapidly as possible while the eccentric phase was controlled. For all loads, main effects of age and gender were noted with greater peak power in young participants and in men. Further, there was trend for older men to decline in maximum concentric power at a faster rate than the other groups when working against loads greater than 40% of maximum isometric contraction force. In terms of peak velocity during the knee extension power test, a significant main effect of age but not of gender was observed at each load with the shortening velocity being higher in young participants at all loads. This indicates that age-related losses of muscle power are primarily driven by impairments in explosive contractile velocity [21]. 

In order to elucidate the influence of muscle mass on the age-related loss in muscle power, Thom et al. [32] measured triceps surae power and volume using an isokinetic dynamometer and a magnetic resonance imaging scanner in young (age 19 to 35 years) and older healthy men (age 69 to 82 years). Peak power was markedly reduced in the older as compared to the younger men (45%). In addition, older subjects exhibited 81% of the younger subjects muscle volume. Further, when muscle power was normalized to muscle volume, the so-called specific power (i.e., power/volume) was 55% lower in old compared to young men. This result illustrates that only approximately half of the loss in triceps surae peak power in old age is due to decreases in muscle volume. Thus, other neuromuscular factors have to be taken in account. In fact, Häkkinen et al. [22] observed significantly lower activities of the m. vastus lateralis/medialis during two concentric power-related test conditions (i.e., standing long jump, high-velocity leg-extensor contraction at 50% of the individual 1RM) in old (age 62 to 78 years) versus middle-aged men and women (age 35 to 45 years) using surface electromyography. In addition, antagonist muscle activities (i.e., m. biceps femoris) during power performances were significantly greater than the corresponding antagonist activation recorded during isometric action [22]. 

In summary, muscle power peaks between the ages of 20 to 30 years and declines after the age of 65 years at a fast rate. The loss in muscle power is specifically pronounced in the ninth decade of life. Men appear to be equally or even more affected by power loss than women. Lower muscle volume and an impaired ability to activate muscles appear to be responsible for the age-related decline in muscle power.

### 2.2. Age-Related Effects on Measures of Balance

Balance can be described as the ability to control the body's position in space for the purpose of balance and orientation [33]. Under static conditions, the base of support (i.e., feet) and the ground (i.e., surface in treadmill walking) remain stationary and only the centre of mass moves, whereas under dynamic conditions, both, the base of support and the centre of mass shift [34]. Different balance strategies have been identified that help keeping the centre of mass over the base of support. Shumway-Cook and Woollacott [33] differ between a proactive (i.e., anticipation of a predicted disturbance), a reactive (i.e., compensation of a disturbance), and a static/dynamic steady-state (i.e., maintaining a steady position in sitting, standing, and walking) balance strategy. Given that a large number of falls occur during ambulation (i.e., steady-state balance) in the elderly [35] or during slipping and tripping events (i.e., reactive balance) [36], the focus will be laid on the age-related effects affecting dynamic steady-state and reactive balance.

#### 2.2.1. Steady-State Balance

The ability to control posture is a dynamic process across the life span. There is evidence that young children and older adults show the largest magnitudes of postural sway and the slowest gait speeds. Therefore, a U-shaped dependency between variables of static steady-state balance and age (i.e., sway velocity) [37] and an inverted U-shaped dependency between measures of dynamic steady-state balance and age (i.e., gait speed) [38] can be postulated. 

Era et al. [39] assessed performance in normal, semitandem, and tandem stance on a force platform in a randomly selected sample of subjects aged 30 years and over. They observed that deterioration of the postural control mechanisms starts relatively early in life. Differences in balance performance were already apparent among young (30 to 39 years old) and middle-aged adults (40 to 49 years old) and became even more pronounced after the age of 60 years. 

In another study, Colledge et al. [41] investigated postural sway under 4 test conditions (i.e., on firm surface with eyes open, on firm surface with eyes closed, on a foam surface with eyes open, and on a foam surface with eyes closed) in 4 different age groups (i.e., 20 to 40 years, 40 to 60 years, 60 to 70 years, and >70 years). Sway increased linearly with age but was not affected by gender. Further, it was found that subjects in all age groups relied more on proprioceptive than on visual input. Of note, dependence on vision and proprioception did not alter with advancing age [41]. 

When investigating young (mean age 20 years) and older healthy adults' (mean age 70 years) ability to control posture under conditions of increasing task complexity (i.e., normal quiet bipedal stance, sharpened or tandem Romberg stance, one-legged stance on the dominant leg), Amiridis et al. [42] observed an increase in postural sway as a result of narrowing the base of support in both groups. However, greater centre of pressure excursions, muscle activities, and joint displacements was found in old compared to younger adults. Further, older adults displayed increased hip movement accompanied by higher hip muscle activity, whereas no similar increase was noted in the younger group. The authors concluded that the older adults rely more on a hip strategy as posture is challenged by increased task constraints during quiet standing [42].

In terms of the dynamic component of steady-state balance, Callisaya et al. [43] recently studied the effects of aging on temporal and spatial gait variability measures (i.e., step time, step length, step width) in healthy adults aged 60 to 86 years. Older age was associated with greater variability in all gait measures. All relationships were linear, except that between age and step time variability, which was curvilinear in women.

**Figure 2:**
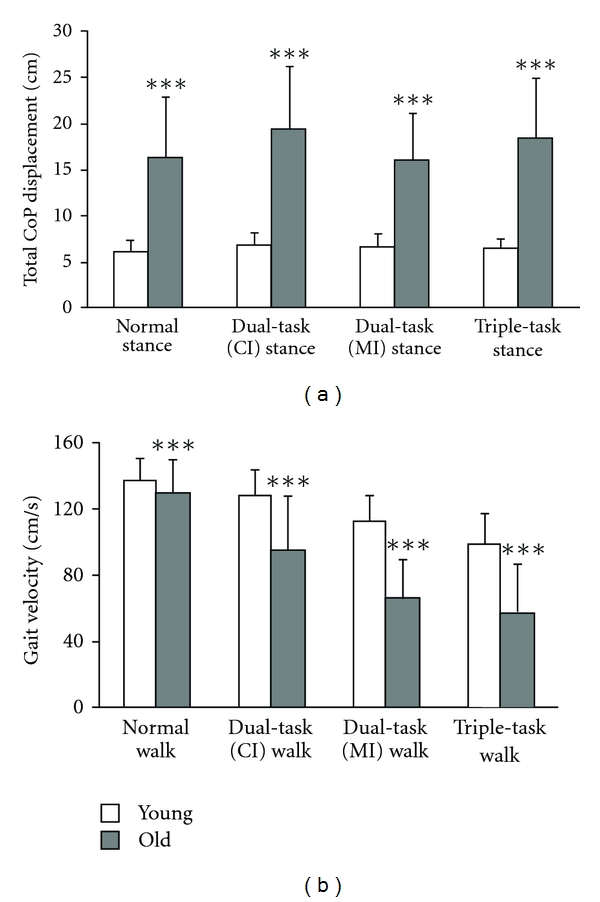
Age-related differences in normal and multitask balance performance. (a) CoP displacement during standing. (b) Gait velocity during walking. Young different from old: ***P < 0.001. CoP: center of pressure, CI: cognitive interference task, MI: motor interference task. Adapted from Granacher et al. [18, 40].

For many years, the control of posture was solely attributed to automatic or reflex controlled muscle activations [44]. However, today it is well-known that attentional resources are necessary to effectively stabilize the body's centre of gravity over the base of support [45]. One form of investigating the attentional demands in postural control has been the application of dual-task paradigms. Granacher et al. [18, 40] recently examined the effects of a cognitive (i.e., serial subtractions by three) and/or a motor interference (i.e., holding two interlocked sticks steady in front of the body) task on postural sway (i.e., standing on a balance platform), gait velocity/variability (i.e., walking on an instrumented walkway) in young (mean age 22 years) and elderly healthy subjects (mean age 73 years). Irrespective of the task condition, that is, single or multitask, elderly participants showed larger displacement of the centre of pressure, slower gait velocity, and greater stride-to-stride variability than younger participants (Figures 2(a) and 2(b)). Further, in both age groups, postural sway and stride-to-stride variability increased and gait velocity decreased with progression in task complexity [18, 40]. The authors speculated that greater postural sway/gait decrements during the concurrent performance of attention demanding tasks are probably due to age-related deteriorations in the postural control system and the inability to allocate attention properly between steady-state balance and a cognitive and/or motor interference task [18, 40]. 

A number of theories have been proposed to explain dual-task interference effects [46, 47]. First, according to capacity theories, task performance suffers because both tasks have to compete for, or somehow share, a finite pool of cognitive resources. Second, bottle-neck theories propose that performance suffers because both tasks have to queue up to use a single critical information-processing channel. Third, cross-talk theories propose that the processing of one task creates noise that interferes with performance of the second task. Finally, neural structure theories propose that dual-task interference effects occur because there are competing demands for specific neural pathways within the brain. 

Further evidence for the involvement of supraspinal structures in the control of stance and gait comes from studies using magnetic resonance imaging. In an attempt to investigate supraspinal mechanisms responsible for age-related changes in gait characteristics, Rosano et al. [48] assessed gray matter volume of 5 different brain regions and spatiotemporal gait parameters in older adults with a mean age of 78 years. Shorter steps and longer double support times were associated with smaller sensorimotor regions within the motor, visuospatial, and cognitive speed domains. These findings suggest that measures of gait in older adults living in the community are not only the consequence of underlying age-related changes in peripheral systems (i.e., neuropathology) [49], but that they also indicate underlying focal, selective changes in brain structure [48].

In summary, older adults show larger postural sway, slower gait velocity, and increased stride-to-stride variability under single and particularly multi-task conditions compared to young adults. During quiet standing, older adults appear to compensate for greater instability by applying different balance strategies (i.e., hip strategy) and by increasing muscle activity. Age-related changes in the gait pattern are most likely caused by degenerative processes in the peripheral and the central nervous system.

#### 2.2.2. Reactive Balance

Slips and trips account for 30% to 50% of falls in community-dwelling older adults [50]. Therefore, many researchers investigated age-related changes in balance recovery mechanisms. In fact, Lin and Woollacott [51] determined postural muscle response characteristics following various sizes of support surface perturbations in young (mean age 25 years), stable older (mean age 73 years), and unstable older adults (mean age 76 years). Slower onset latencies, smaller magnitudes of postural responses, and longer maintenance of postural muscle activation were found in response to platform perturbations in both stable and unstable older subjects compared to young adults. Whereas delays in onset times and smaller amplitudes of muscle responses can be classified as age-related deteriorations in postural control, the prolonged muscle activation might be a compensatory mechanism to help preserve postural stability [51]. Notably, unstable older adults were not able to show this compensatory mechanism in all test conditions in contrast to stable older adults. 

In a more functional approach, Tang and Woollacott [52] investigated postural responses to unexpected forward slips during walking in young adults (mean age 25 years) and active older adults (mean age 74 years). A similar activation sequence of postural muscles in response to accelerating perturbation impulses in young and elderly subjects was observed. However, postural responses of older adults were of longer onset latencies, smaller magnitudes, and longer burst durations compared to young subjects. 

**Figure 3:**
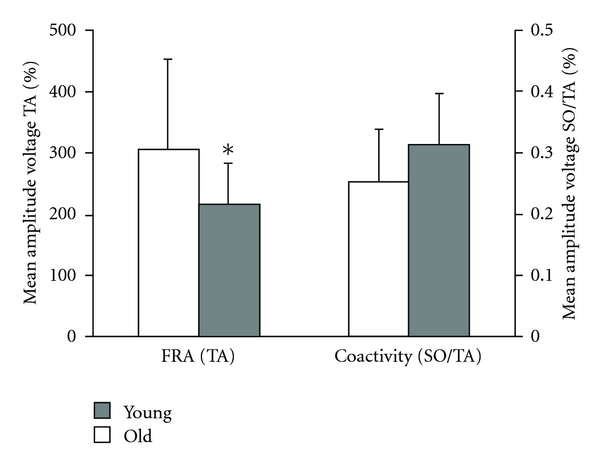
Age-related differences in reactive balance performance (i.e., perturbed walking). FRA: functional reflex activity, SO: m. soleus, TA: m. tibialis anterior. Young different from old: *P < 0.05. Adapted from Granacher et al. [15].

Recently, Granacher et al. [15] investigated postural responses to unexpected decelerating gait perturbations during walking on a treadmill in young (mean age 27 years) and elderly healthy men (mean age 67 years). The authors observed significantly smaller magnitudes in reflex activity of the prime mover compensating for the perturbation impulse and a tendency towards a higher level of coactivation in muscles encompassing the ankle joint (Figure 3). These inefficient balance strategies seem to make older adults more prone to falling compared to young adults. In accordance with this hypothesis, Pavol et al. [53] identified delayed support limb loading (>145 ms) in response to an external perturbation as a deficit that increases the risk of falling. 

In summary, older adults show deficits in the compensation of perturbation impulses during standing and walking. Slower onset latencies, reduced reflex activities, increased antagonist coactivations, and longer burst durations of muscles compensating for stance/gait perturbations were reported for old compared to young adults.

## 3. Associations between Measures of Muscle Strength/Power and Balance Performance

From a therapists' or practitioners' point of view, knowledge about the relationship within the different dimensions of postural control (e.g., steady-state balance, reactive balance) and muscle strength (i.e., isometric and dynamic muscle strength) as well as between postural control and muscle strength/power is important for both the identification of persons with an increased fall risk and the development of fall-preventive training programs. More specifically, given the high incidence rate of fall-related injuries in older adults [11], findings on potential associations within the different dimensions of balance and strength as well as between these two neuromuscular capacities could provide scientific rationales to fall-risk assessment as well as to the development of specifically tailored fall prevention and rehabilitation programs in older adults.

### 3.1. Associations between Measures of Isometric and Dynamic Muscle Strength

In terms of isometric and dynamic muscle strength, Knapik et al. [54] investigated in an early study the relationships among isokinetic, isometric, and isotonic (i.e., 1RM) strength measurements in knee and elbow flexion/extension in young healthy men with a mean age of 26 years. Correlations among the 3 testing modes at joint angles of peak isometric torque were generally high (mean: *r* = 0.78; range: *r* = 0.97 to 0.47) for all tested muscle groups. The amounts of common variance suggested that the 3 strength testing modes were measuring a similar phenomenon which they consequently termed maximal voluntary strength [54]. 

Izquierdo et al. [23] followed a similar approach in healthy older adults with a mean age of 65 years. For this purpose, participants performed maximal isometric knee extensions, 1RM knee extensions, 1RM half squats, squat jumps (SJ), and countermovement jumps (CMJ). The authors observed statistically significant correlations between variables of isometric strength (i.e., MIS, RFD) and measures of dynamic strength (i.e., 1RM tests) ranging between *r* = 0.47 and *r* = 0.66 (all *P* < .05). However, no significant associations were found between MIS/RFD and measures of lower extremity power (i.e., jump performance).

In summary, there is an association between selected variables of isometric and dynamic muscle strength in older adults.

### 3.2. Associations between Measures of Steady-State and Reactive Balance

Hsiao-Wecksler et al. [55] studied potential association between measures of steady-state and reactive balance in healthy young (mean age 25 years) and older adults (mean age 69 years). In both groups, significant associations were found between centre of pressure displacements during quiet stance and during mild perturbation. Based on their results, Hsiao-Wecksler et al. [55] concluded that it is possible to predict the dynamic postural control response from quiet stance behaviour in young and older adults. Therefore, the authors suggested that the postural control system may use the same control mechanisms during quiet stance and mild perturbation conditions. 

Shimada et al. [56] investigated steady-state (i.e., sensory organization test) and reactive balance (i.e., decelerating perturbation impulse while walking on a treadmill) in healthy young (age 20 to 32 years) and older adults (age 65 to 79 years). Only weak but nonsignificant correlations were found in the elderly subjects for measures of standing balance and balance recovery during the compensation of the perturbation impulse. The reason for the discrepancy between the results of Shimada et al. [56] and the findings of Hsiao-Wecksler et al. [55] is most likely related to the different methods applied in these studies. In the study of Shimada et al. [56], associations between quiet stance measures and measures of gait perturbation were investigated, whereas Hsiao-Wecksler et al. [55] examined the relationship between measures of quiet stance and measures of mild perturbation during standing. Hsiao-Wecksler et al. [55] reported that the compensation of these perturbation impulses did not even force subjects to take a step for the maintenance of balance. Thus, it can be speculated that in fact different neuromuscular mechanisms might be responsible for the regulation of a primarily static/steady-state postural control task (e.g., quiet stance, mild stance perturbation) and a dynamic/reactive postural control task (e.g., gait perturbation). This hypothesis is strengthened by findings from Kang and Dingwell [57] who examined the relationship between postural stability during quiet stance and locomotor stability during walking on a treadmill in healthy adults with an age range of 18 to 73 years. The authors found that standing and walking exhibited local dynamic stability properties that were significantly different and not correlated [57]. 

In another study, Granacher et al. [18] assessed the relationship between quiet stance and walking under multi-task conditions in a cohort of young (mean age 22 years) and elderly healthy subjects (mean age 73 years). No significant associations were detected between measures of quiet stance and walking under multi-task conditions. Thus, it appears that the mechanisms governing standing and walking stability under single and multi-task conditions are significantly different [18, 57].

In summary, measures of steady-state and reactive balance as well as variables of static and dynamic steady-state balance under single and multi-task conditions appear to be unrelated.

### 3.3. Associations between Measures of Strength/Power and Balance Performance

In a recent study, Granacher et al. [15] examined whether there is a relationship between measures of isometric strength of the leg extensors and variables of reactive balance in young (mean age 27 years) and elderly healthy men (mean age 67 years). No significant correlations were found between MIS/RFD of the leg extensors and functional reflex activity during the compensation of a gait perturbation impulse indicating that different mechanisms regulate these neuromuscular capacities.

Ringsberg et al. [58] scrutinized the relationship between measures of steady-state balance (e.g., one leg standing balance, gait speed) and maximal isometric knee extensor/flexor and ankle dorsiflexor strength in 75-year-old women. Neither of the muscular strength tests was related to the one-leg standing test. However, all muscular strength tests were highly associated with gait speed.

In another study, Buchner et al. [59] measured peak torque of the knee (i.e., extension/flexion) and the ankle (i.e., plantar/dorsiflexion) as well as gait speed (i.e., steady-state balance) in a sample of healthy adults aged 60 to 96 years. Notably, the authors found significant associations between lower extremity strength and gait speed in frail/weak subjects whereas there was no association in nonfrail/strong subjects [59].

In a more functional approach, Bean et al. [60] assessed the influence of leg power and leg strength on physical performance (i.e., tandem gait, stair time, chair-stand time, gait velocity, short physical performance battery) in community-dwelling mobility-limited older people aged 65 to 83. Although leg power and leg strength were strongly correlated (*r* = 0.89); leg power was recognized as a separate attribute that exerted a greater influence on physical performance. In fact, leg power modelled up to 8% more of the variance of the physical performance measures.

In summary, there is a relationship between gait speed (i.e., dynamic steady-state balance) and measures of isometric/dynamic strength and power particularly in frail older adults. However, one-leg standing balance (i.e., static steady-state balance) and the ability to recover from gait perturbations (i.e., reactive balance) are not associated with measures of isometric strength.

## 4. Resulting Consequences for the Assessment of Strength and Power

The reported associations between variables of isometric strength, muscle power, and jump performance [23, 54] indicate that these strength-testing modes are measuring a similar phenomenon [54]. Based on these findings, it can be argued that it is sufficient to either test MIS or muscle power. However, given that the age-related loss of muscle power occurs at a faster rate than muscle strength [13] and that power producing capabilities are more strongly associated with functional performance than muscle strength [60], it is recommended to particularly include the analysis of lower extremity muscle power in a standard strength/power assessment protocol for older adults. However, as of now, this is not supported by predictive data. A sophisticated but still easy-to-administer time efficient and at the same time a rather cost effective test for the assessment of leg extensor power is the application of the SJ and/or CMJ on a force plate. Over the last years, the analysis of plyometric tests using force plates became user friendly and the parameter power is usually integrated as a default measure in the data report which makes this test even suitable for a clinical setting.

In summary, the analysis of muscle power should be incorporated in a standard fall-risk assessment protocol due to its functional relevance. Plyometric tests on force plates (i.e., SJ, CMJ) are feasible, safe, time efficient, and valid test instruments. 

### 4.1. Tests for the Assessment of Strength and Power

Strength/power performance can be assessed using a variety of contraction modalities (i.e., isometric, concentric/eccentric, isokinetic) and methods (i.e., weight machines/free weights, force plates, isokinetic dynamometers, etc.).

#### 4.1.1. One or Multiple Repetition Maximum

A well-accepted and easy-to-administer test is the so-called one repetition maximum (1RM) test [61]. The 1RM is defined as the heaviest load an individual is able to lift only once through a full range of motion on a weight machine or with free weights. Ideally, it is determined within 3 to 5 attempts. The American College of Sports Medicine (ACSM) provides guidelines for 1RM tests [61]. Alternatively, multiple RM tests are often applied in a geriatric context to avoid test-induced injuries due to maximal contractions. Using multiple regression analyses, 1RM strength can be predicted from multiple RM testing, anthropometry, gender, age, and training history. Reynolds et al. [62] provided exercise-specific prediction equations for the 1RM. The authors concluded that the most accurate prediction of strength occurred from a 5RM test, with the accuracy of prediction worsening with increasing repetitions to failure (10RM, 20RM).

#### 4.1.2. Maximal Isometric Strength (MIS) and Rate of Force Development (RFD)

**Figure 4:**
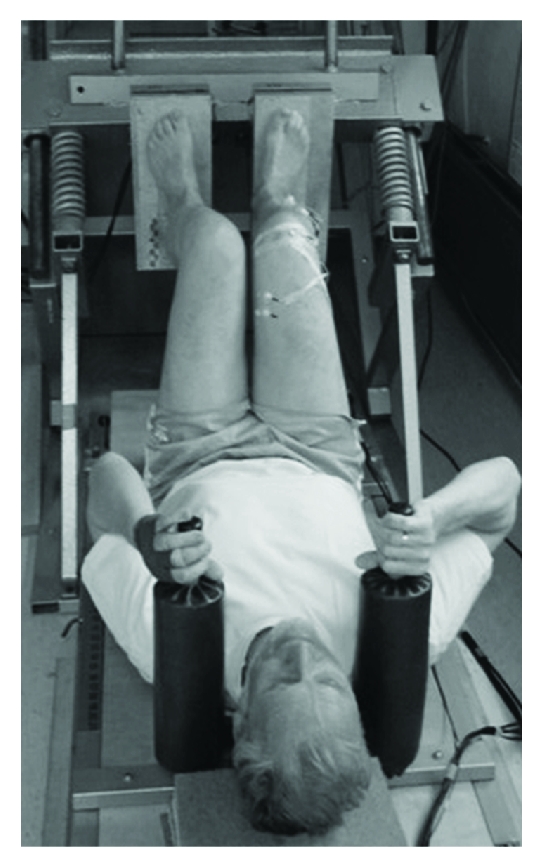
Assessment of maximal isometric strength and rate of force development using a leg press (i.e., force signals will be recorded by a separate force plates underneath the feet).

Strength can be defined as the ability to produce force [63]. Isometric strength testing is characterized by maximal contractions against an immovable resistance. Thus, the measurement of MIS is simplified because variables like velocity and muscle length are kept constant. Given that leg-extensor strength is associated with functional performance (e.g., maximal gait velocity) in older adults [60], it is of interest to analyse MIS and RFD of the leg extensors. For testing purposes, custom-built force plates (e.g., AMTI, KISTLER, etc.) are often integrated in weight machines to determine MIS and RFD of the leg extensors (Figure 4). MIS is defined as the maximal voluntary force value of the force-time curve, determined under isometric condition. RFD is defined as the maximal slope at deflection of the force-time curve (Δforce/Δtime) [15] or as the mean slope of the force-time curve between 20% and 80% of the individual maximal force under isometric condition [64]. The latter procedure appears to be more robust regarding movement artefacts (i.e., kicking of the heel at the onset of contraction) than the calculation of maximal slope RFD. Further, it was recommended to additionally determine the mean slope of the force-time curve over the time intervals 0–30 ms (RFD_30_) and 0–100 ms (RFD_100_) after onset of force [15]. Of note, age-related deficits in force production can particularly be observed in these early intervals of the force-time curve [15].

#### 4.1.3. Muscle Power

Power production is defined either as work divided by the time over which it is completed or as the force/torque of a muscular contraction multiplied by its velocity [65]. In general, isokinetic dynamometers and/or vertical jumping protocols on force plates (e.g., CMJ) are usually used to assess muscle power.

**Isokinetic Tests**

Isokinetic tests are characterized by a constant angular velocity over the full range of motion which is independent of the contraction intensity. As a function of the tested muscle, angular velocities usually range between 30°/s and 240°/s. Isokinetic power is calculated as a product of the peak torque (Nm) at a specific velocity and the respective angular velocity (°/s). However, a disadvantage of isokinetic testing protocols is that isokinetic movement does not adequately reflect natural human movement behaviour as demanded in actual human performance tasks.

**Tests on a Force Plate**

The application of plyometric tests on a force plate mainly comprises SJs and/or CMJs in a geriatric context. An SJ is characterized by a semisquatted start position with no countermovement that is followed by an explosive concentric vertical upward movement, resulting in a maximal vertical jump. During the CMJ, subjects stand in an upright position on the force plate and are instructed to begin the jump with a downward movement, which is immediately followed by a concentric upward movement, resulting in a maximal vertical jump. Peak power is analysed during the push-off phase of the SJ/CMJ by integrating the force-time record. If force plates are not available, equations reported in the literature can be used to calculate power from jump height, body mass, and/or body height [66].

A more functional test for the assessment of leg extensor muscle power is the sit-to-stand transfer test. This test requires participants to sit on the front part of a chair with arms crossed in front of the chest, with the gaze fixed straight ahead, and with both feet placed on a force plate. The participants are then asked to rise as fast as possible into the standing position and to stand quietly for 5 s. According to Lindemann et al. [67], power is calculated using the changes in vertical ground reaction force during the rising phase, vertical ground reaction force during quiet standing, and the difference in body height during the sitting and standing position. Recently, Bohannon [68] conducted a systematic literature review regarding test-retest reliability of the sit-to-stand test and found moderate to excellent intraclass correlation coefficients ranging from 0.64 to 0.96. In addition, Zech et al. [69] observed that the assessment of leg extensor power during the sit-to-stand test is a sensitive marker to distinguish between community-dwelling nonfrail and prefrail older adults.

In summary, one or multiple RM tests are safe and easy to administer. They are particularly suitable for the determination of training intensity during a conditioning program. The assessment of MIS, RFD, and muscle power requires sophisticated testing equipment (i.e., force plate, isokinetic dynamometer, etc.) but provides detailed information on force and power production in laboratory (i.e., CMJ) and more functional situations (i.e., sit-to-stand test) which could be helpful to identify older adults being at risk of future functional limitations [13].

## 5. Resulting Consequences for the Assessment of Balance

Given that falls primarily occur during ambulation and not during quiet standing in the elderly [70] and that standing balance (i.e., static steady-state balance), walking balance (i.e., dynamic steady-state balance), and balance recovery (i.e., reactive balance) were reported to be unrelated [18, 56, 57], fall-risk assessment should particularly be carried out under dynamic steady-state balance (e.g., analysis of gait variability under single and particularly multi-task conditions) and reactive balance conditions (e.g., exposure to balance threats via the postural stress test or platform translations during sit-to-stand tasks) to identify older adults at risk of falling.

For a clinical setting, the “stops-walking-when-talking test” [71] as well as the postural stress test [72] appears to be well-suited for the identification of older adults at risk of falling because they are easy to administer and provide immediate test results. More sophisticated monitoring of older adults' fall risk is usually conducted with the help of instrumented and pressure-sensitive gait mats (e.g., GAITRITE) or corridors of photoelectric cells (e.g., OPTOGAIT). Irrespective of the test system, standard gait analysis parameters (e.g., gait speed, cadence, step/stride length/time, step/stride length/time variability, percent stance phase, percent swing phase, percent single support phase, etc.) are immediately available after the tests are completed. Guidelines for instrumented gait analysis in older adults were presented by Kressig et al. [70].

In summary, tests for the analysis of steady-state and reactive balance should be incorporated into a standard fall-risk assessment protocol for older adults. Easy-to-administer clinical tests are the “stops-walking-when-talking test” and the postural stress test. The inclusion of a gait analysis using instrumented walkways is highly recommended to obtain important gait parameters (i.e., gait variability particularly under multi-task conditions) that are strongly associated with fall risk in older adults.

### 5.1. Tests for the Assessment of Balance

In general, balance can be tested using a variety of clinical (e.g., gait speed), biomechanical (e.g., pressure-sensitive walkway), and electrophysiological tests (e.g., electromyography). Kapteyn et al. [73] provided recommendations for posturographic testing. Briefly, different factors (e.g., room illumination, temperature, noise, defined test positions, test instructions, etc.) have to be considered to obtain standardized test circumstances. Due to the journal's space limitations, only a small selection of clinical and laboratory tests will be presented in the following. For a comprehensive review on this topic, the reader is referred to the work of Yim-Chiplis and Talbot [74].

#### 5.1.1. Steady-State Balance

**Figure 5:**
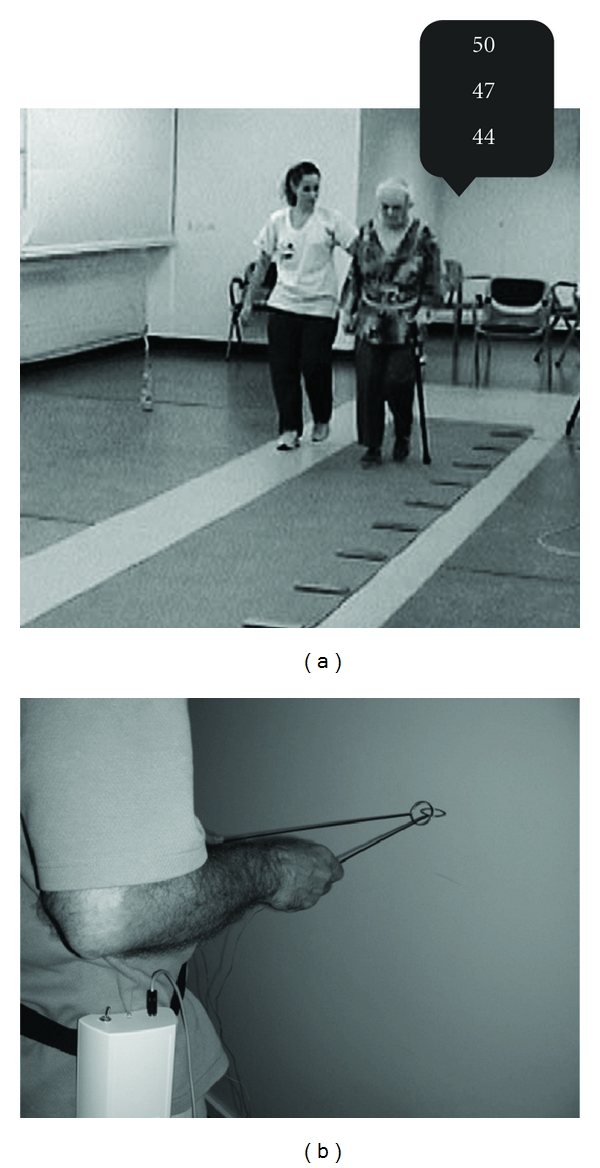
Assessment of steady-state balance performance. (a) 10 m walk test with concurrent cognitive interference task (i.e., counting backwards by three). (b) 10 m walk test with concurrent motor interference task (i.e., holding two interlocked sticks steady in front of the body).

Steady-state balance can be assessed during standing and/or walking under single-task conditions (i.e., standing/walking only) and/or dual/multi-task conditions (i.e., standing/walking while concurrently performing a motor/cognitive interference task) (Figures 5(a) and 5(b)).

**Clinical Tests**

One-leg standing balance (i.e., ability to stand unassisted for 5 seconds on one leg) is an easy-to-administer and inexpensive clinical test for the assessment of the functional level and the frailty status of older community-living persons [75]. Notably, Vellas et al. [76] reported that this test can be used as a predictor of injurious falls.

The “Timed Up and Go Test” (TUG) is a test of dynamic steady-state balance that is commonly used to assess functional mobility and risk of falling in community-dwelling, frail older adults (aged 70 to 84 years) [77]. The test requires subjects to stand up from a 44 to 47 cm high chair without using the arms, walk 3 m, turn, walk back, and sit down. Excellent interrater reliability (*r* = .99) and moderate test-retest reliability (*r* = .56) were reported for the TUG [77, 78]. Further, a time >14 s to complete the test differed between older fallers and nonfallers [79]. Recently, Beauchet et al. [80] found that the imagined TUG or iTUG (i.e., time needed to imagine performing the TUG) is clinically feasible among frail older adults and that it proved to be a useful tool as a marker of balance and gait disorders in older adults with a mean age of 85 years.

The measurement of gait velocity (e.g., time required to walk) is a simple and inexpensive test that can be used in a clinical setting to detect mobility problems [81] and to predict adverse outcomes (i.e., hospitalizations, new falls, and requirement for a caregiver) in healthy seniors aged 75 and older [82]. The functional implications of gait velocity have frequently been described and discussed in terms of the time that is needed to cross a street safely. In fact, Hoxie et al. [83] found out that a mean gait speed of 122 cm/s is required to cross a street during a green light period. The same study revealed that 96% of pedestrians aged 65 and over walk with a gait velocity slower than 122 cm/s.

In addition, Guimaraes and Isaacs [84] found slower gait speeds in elderly people aged 65 and over who were admitted to hospital shortly after suffering a fall compared with patients of similar age admitted to the same hospital who had not suffered a recent fall. In addition, with the help of an easy-to-administer test, Lundin-Olsson et al. [71] observed that elderly subjects who stopped walking when talking had a significantly increased risk of sustaining a fall within the next six months.

**Biomechanical Tests**

Using biomechanical testing equipment (e.g., force plates), postural sway (i.e., centre of pressure displacements) can be analysed during bipedal stance, step stance, tandem stance, or one-legged stance, with eyes opened or closed, on stable or unstable (e.g., balance pad) ground, under single or multi-task conditions. Fernie et al. [85] investigated healthy subjects aged over 63 years and observed that postural sway during bipedal stance with eyes opened and closed (i.e., mean sway speed) was significantly greater for those who fell one or more times in a year than for those who did not fall. Using different postural sway measures, Tucker et al. [86] were recently able to identify community-dwelling older adults with a fall history.

Hausdorff et al. [87] were among the first to investigate that gait unsteadiness in terms of greater temporal and spatial stride-to-stride variability significantly differed between healthy older community-dwelling fallers (mean age 82 ± 5 years) and nonfallers (76 ± 4 years). For this purpose, coefficients of variation (CV) were calculated for stride and swing time, stride length, and stride width according to the following formula: [(SD/Mean)∗100] [70]. The smaller the CV value, the better the walking pattern. Besser et al. [88] reported that 5 to 8 strides are necessary for 90% of individuals tested with a pressure-sensitive walkway (i.e., GAITRITE) to have reliable mean estimates of spatiotemporal gait parameters. Recently, Hollman et al. [89] presented normative spatiotemporal gait parameters (e.g., stride time/length, stride time/length variability, gait speed, cadence, etc.) in older men and women that can be used to identify subjects with gait disorders. Moreover, Kressig et al. [90] were able to identify critical thresholds for stride time CV under single- (i.e., walking only, >4%) and dual-task conditions (i.e., walking while concurrently counting backwards, >10%) that were strongly associated with fall events in older inpatients. In addition, a recent systematic review on dual task performance and the prediction of falls indicated that changes in performance whilst dual-tasking were significantly associated with an increased risk for falling amongst older adults [20].

#### 5.1.2. Reactive Balance

**Figure 6:**
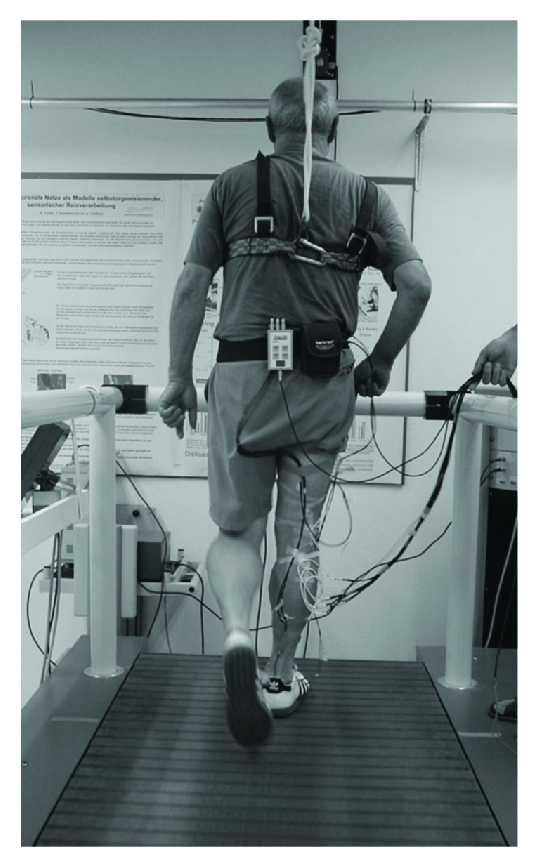
Assessment of reactive balance performance using a motorized treadmill (i.e., unexpected decelerating gait perturbations during walking will be applied).

Reactive balance can be tested with a wide variety of easy-to-administer clinical tests (e.g., postural stress test, nudge test) or more sophisticated biomechanical (e.g., stance/gait perturbations on force platforms or treadmills, Figure 6) and electrophysiological testing equipment (e.g., electromyography, h-reflex).

**Clinical Tests**

The sternal shove test or nudge test is a simple test of balance recovery [91]. Subjects stand with feet close together. The examiner pushes with light even pressure over the sternum three times. The response is graded using a 0 to 2 scale with 0 meaning that the subjects start to fall and need assistance; 1 indicates that the subject maintains balance with feet movement; 2 means that the subject's stance remains stable. However, reliability and validity have not been established for this test [92]. This might be due to the fact that the intensity of the applied perturbation impulse as well as the rating of the balance recovery reaction is examiner dependent.

A more sophisticated but still easy-to-administer test for the assessment of balance recovery reactions is the so-called postural stress test which was introduced by Wolfson et al. [72]. In this test, balance recovery reactions to postural perturbations of varying degrees are measured during normal standing using a simple pulley weight system that displaces the centre of gravity behind the base of support. More specifically, subjects have to withstand a series of posterior perturbation impulses that are applied at the level of the subject's waist using three different perturbation intensities (i.e., 1.5%, 3%, and 4% of the body mass). Scoring of the postural responses is based on a nine-point ordinal scale, where a score of 9 represents the most efficient postural response and a score of 0 represents a complete failure to remain upright [93]. Chandler et al. [93] observed that elderly community-dwelling fallers score significantly lower on the postural stress test than either young adults or nonfalling elderly individuals.

**Biomechanical Tests**

Biomechanical tests are usually characterized by high criterion validity. However, laboratory tests are generally expensive, complex, and time consuming which is why they are primarily suitable for research purposes and not for clinical practice.

In an earlier study, Maki et al. [9] compared the ability of different measures of postural balance to predict risk of falling prospectively in an ambulatory and independent elderly population aged between 62 and 96 years. Different balance tests including tests of spontaneous sway, induced sway, and one-legged tests were conducted. A force plate moving back and forth and side to side was used during the induced sway conditions. A number of measures showed evidence of significant differences between fallers and nonfallers. The differences were most pronounced for measures related to the control of both spontaneous and sway-induced lateral stability. The authors suggested that this rather simple and safe force-plate measure of postural sway can be used in a clinical setting as a preliminary screening tool for risk of falling [9].

In a more recent study, Pavol et al. [94] investigated fall incidence in old compared to young healthy subjects when confronted with unexpected slips during a sit-to-stand task. Trials began with subjects sitting on a stool in a standardized position with their feet resting on horizontally moveable force platforms. Subjects' task was to stand up as quickly as possible without using their arms and to remain standing still. After four normal sit-to-stand trials, a slip was induced without warning when the stool supported less than 10% of the subjects' body mass. The authors observed that older adults with a mean age of 73 years were more likely to fall upon initial, unexpected perturbation exposure. In fact, 73% of the older adults fell upon the first slip whereas only 28% of the young adults fell [94].

Pijnappels et al. [19] induced trips through obstacles that unexpectedly appeared from the ground while subjects were walking at a self-selected speed over a platform. Tripping reactions were applied at midswing, corresponding to 40% of the normal swing phase duration for all subjects. Kinematic data, ground reaction forces, and centre of pressure of the support limb were analysed in this study. It was found that particularly older subjects who fell in this tripping experiment showed insufficient reduction of the angular momentum during push-off and less proper placement of the recovery limb compared to older nonfallers and young subjects [19].

A limitation of a large number of reactive balance studies is that they usually investigate postural responses during standing or walking in young versus older adults. However, studies using a retrospective (i.e., fallers versus non-fallers) or even prospective design (i.e., identification of fallers with the help of laboratory-based balance recovery reactions) are rare. For instance, Smith et al. [95] investigated long latency ankle responses to dynamic perturbation in older adults and could not find significant differences between fallers and non-fallers in latencies or magnitudes of reactive lower extremity muscle responses. Recently, Pai et al. [96] examined older community-dwelling adults' (>64 years) future fall risk and their reactive responses and adaptations to repeated slips. Experimental slips were induced at seat-off during a sit-to-stand task by a computer-controlled release of two sliding platforms. Each slip outcome was scored as 0 (successful recovery), 1 (loss of balance), and 2 (fall). The slip outcome scores for 7 trials were summed for each participant and identified as the slip score, ranging from 0 to 14. Approximately 30 months after the initial laboratory investigation, self-reported falls data were collected for the preceding year. The authors found that a higher overall slip score or having lost balance during the second reslip trial was associated with greater likelihood of future falls. However, the findings of this study have to be interpreted as preliminary due to the small sample size applied in this investigation (N = 13). A post-hoc power analysis revealed that 200 participants would have been needed to provide adequate statistical power for the prediction of healthy older adults' annual fall risk. Therefore, further studies have to be conducted to fill the gap between findings from laboratory-based reactive balance studies and the epidemiology of falls.

In summary, the assessment of steady-state and reactive balance is easy to administer with the help of clinical tests. Large populations of older adults can be monitored in terms of mobility limitations and risk of falling. However, sensitivity (i.e., the proportion of true positives that are correctly identified by the test) and specificity (i.e., the proportion of true negatives that are correctly identified by the test) of clinical tests are limited which is why, especially in research settings, more sophisticated biomechanical testing equipment is applied for monitoring older adults' balance performance. Given that falls often occur during ambulation, it was proposed that particularly dynamic steady-state and reactive balance measures are useful for screening purposes. During the last years, gait variability especially under dual-task conditions was recognized as a sensitive marker for the identification of older adults with a risk of falling.

## 6. Resulting Consequences for Strength and Power Training

The above reported findings on the relationships between the different strength modes [23, 54] together with the results on the associations between leg extensor strength/power with functional performance [60] have meaningful implications for the application of adequate and effective resistance training programs.

The implementation of traditional heavy-resistance training protocols can still be recommended, particularly if the goal is to improve strength performance and to induce muscle hypertrophy. In fact, a systematic review of well-designed studies substantiated that resistance training is effective in increasing strength and muscle mass in older adults, with high-intensity training (i.e., 80% of the 1RM) and longer training periods (>12 weeks) being more effective than low-intensity training and shorter training periods [97]. Recently, detailed guidelines for heavy-resistance strength training with older adults were reported in terms of training volume and intensity [98]. A duration of at least 12 weeks, a frequency of 3 times per week, 3 to 4 sets, 8 to 12 repetitions, and an intensity equal to 80% of the 1RM were suggested [98]. 

However, the literature indicates that heavy-resistance strength training increases strength but has less clear effects on balance abilities [97]. In fact, it was shown that 13 weeks of heavy-resistance strength training with 3 training sessions per week had an impact on MIS and RFD in elderly men [99] but not on the ability to compensate for platform [99] or gait perturbations [100]. In addition, a systematic review of randomized controlled trials on the efficacy of resistance training on balance performance could not detect a clear effect of resistance training on various measures of standing balance in older adults (effect size = 0.11) [97]. This rather limited adaptive potential of traditional resistance training restricted to variables of strength could be the reason why no strong effects on functional performance and fall prevention were shown for resistance training alone [101]. Therefore, other resistance training modalities had to be taken into consideration that may have an impact on both, strength and functional performance in older adults. A recent systematic review indicates that resistance training combined with modified power type of exercises or even high-speed power training/ballistic strength training seems to have a greater impact on explosive force production and functional performance in old age than traditional heavy-resistance strength training [102]. 

However, given that the effects of power training on strength and functional performance in older adults are still an emerging field in geriatric research, clear dose-response relationships are lacking. In this regard, de Vos [103] reported that 8 to 12 weeks of power training with high loads (i.e., 80% of the 1RM) induced larger gains in muscle power, strength, and endurance than power training with medium (i.e., 50% of the 1RM) and low loads (i.e., 20% of the 1RM). However, Orr et al. [104] observed that power training at low intensities (i.e., 20% of the 1RM) induced significantly larger improvements in balance performance than power training with medium (i.e., 50% of the 1RM) and high (i.e., 80% of the 1RM) intensities. Significant improvements in peak power, strength, and endurance of lower extremity muscles were observed irrespective of the training intensity [104]. Despite the rather divergent findings, Granacher [98] recommended in a preliminary effort that healthy older adults should perform power training for at least 4 to 6 weeks with 2 to 3 training sessions per week, 1 to 3 sets, and 6 to 12 repetitions using light to moderate resistance (i.e., 40 to 60% of 1RM) with high concentric movement velocities in order to specifically address power capacity.

In summary, the effects of heavy-resistance strength in older adults are restricted to improvements in measures of strength and muscle mass. However, power training or high-velocity strength training has the potential to improve both strength and functional performance. Preliminary data indicates that high-velocity strength training with high loads specifically increases muscle power whereas power training with low to moderate loads improves balance and functional performance.

## 7. Resulting Consequences for Balance Training (BT)

During the last years, numerous studies proved the effectiveness of balance training (BT) on measures of postural control [100], strength [105] and physical performance [106], as well as on fall-incidence rate in older adults [106]. Recently, new trends in BT emerged which produced even larger effects on various measures of balance and physical performance than traditional BT (for a systematic review see Granacher et al. [102]). This is in fact in accordance with the previously reported findings on the nonsignificant associations between different components of balance (static/dynamic steady-state balance versus reactive balance). Based on these results, it appears that the different balance strategies are independent of each other and need to specifically be addressed during intervention programs. In other words, balance exercises comprising steady-state (i.e., static and particularly dynamic exercises under single and especially multi-task conditions) and reactive components (i.e., application of perturbation impulses) should be included in a balance program with the goal to prevent elderly people from falling. It is hypothesized that this new and multifaceted BT program counteracts a larger number of intrinsic fall-risk factors than traditional BT programs. Thus, it may have greater potential to effectively reduce the fall-incidence rate in older adults. However, as of now, there is only preliminary data available that supports this idea. Therefore, future epidemiologic studies need to address this issue in a comparative design to find out whether multifaceted BT regimens are indeed more effective in the prevention of falls than traditional BT programs.

Even though numerous studies investigated the effects of BT in older adults on various measures of balance and functional performance as well as fall rate, clear dose-response relationships are still lacking. Therefore, further research is needed to establish effective training loads and volume for BT. In a preliminary attempt, the ACSM [65] provided in a recent position stand on exercise and physical activity for older adults exercise prescription guidelines for BT:

1. include progressively difficult postures that gradually reduce the base of support (e.g., two-legged stand, semitandem stand, tandem stand, one-legged stand),
2. include dynamic movements that perturb the center of gravity (e.g., tandem walk, circle turns),
3. stress postural muscle groups (e.g., heel stands, toe stands), or
4. reduce sensory input (e.g., standing with eyes closed).

In addition, Granacher [98] presented more detailed information on training load and volume during BT with older adults. According to these guidelines, older adults are advised to perform BT for at least 12 to 13 weeks with 2 to 3 training sessions per week, 3 to 8 sets, and an exercise duration of 20 to 40 s to induce improvements in balance and functional performance [98]. In a recent meta-analysis on exercise to prevent falls in older adults, Sherrington et al. [107] found that BT has the greatest effect on reducing falls as compared to other single interventions. Based on their results, the authors recommended that training intensity during BT should be moderate to high with a training duration of at least 2 hours per week on a permanent basis. They further propose that exercise may be undertaken in a group or home-based setting and that strength and walking training can be included in BT. 

In summary, recent but still preliminary evidence indicates that specific steady-state (i.e., walking while concurrently performing a cognitive and/or motor interference task) and reactive balance exercises (i.e., application of perturbation impulses during standing/walking) should be incorporated in BT for older adults to counteract important intrinsic fall-risk factors. However, clear evidence-based dose-response relationships are lacking. It appears that training intensity during BT should be moderate to high with a training duration of at least 2 hours per week on a permanent basis.

## 8. Conclusions

Age-related deficits in maximal and explosive force production as well as in dynamic steady-state balance particularly under multi-task conditions and in reactive balance represent important intrinsic fall-risk factors in older adults. Correlative analyses indicate that variables of static and dynamic steady-state balance, reactive balance, and muscle strength are unrelated and may thus represent independent neuromuscular capacities. This finding has important implications for fall-risk assessment and for the development of adequate and effective fall-prevention programs. In terms of fall-risk assessment, we therefore strongly suggest to include the analysis of (a) muscle power (e.g., sit-to-stand test on a force plate), (b) dynamic steady-state balance under multi-task conditions (e.g., analysis of gait variability on an instrumented walkway), and (c) reactive balance (e.g., postural stress test with a pulley weight system) into a standard test protocol. 

In terms of fall prevention, we suggest to perform a combination of power training/high velocity strength training with multifaceted BT including multi-task and perturbation-based BT because this combinatory training regimen counteracts a large number of intrinsic fall-risk factors.
